# The Eucalyptus Globulus as a Cancer Remedy

**Published:** 1876-06

**Authors:** 


### THE EUCALYPTUS GLOBULUS AS A CANCER REMEDY.

The profession may well look with distrust on alleged "cancer cures," but the journalist must not omit to mention them. The latest is the famous *eucalyptus globulus*. The *Doctor* quotes six cases reported by Prof. Luton, of Reims, in the *Progres Medi-*

*cal*, all successful; and, as Prof. Luton is a respectable practitioner, and the subject most interesting, we give them:

The first case was that of a woman, seventy-eight years of age, suffering from an encephaloid tumor of the breast, in whom the administration of the tincture of eucalyptus was followed by phlegmonous swelling of the tumor, erysipelatous redness of the surrounding skin, mortification of the tumor, and rapid falling off of the eschars, accompanied with fever, lassitude, anorexia, furred tongue, headahce, and delirium. The cicatricial remains of the tumor had a keloid appearance, and represented the stroma of the original disease. In spite, however, of the continued use of the tincture, this mass subesquently increased in size, and began to assume some doubtful signs of malignancy, when Prof. Luton substituted the powdered leaves of the eucalyptus, one gramme daily, for the tincture, and after three or four days the same phenomena of death of the tumor occurred as on the first occasion. The patient is still under observation, and hopes are entertained by the Professor that the case will terminate in complete cure.

The second case was that of a woman, sixty-eight years of age, suffering from symptoms of cancer of the stomach for nine months, and a tumor in the abdomen as large as a turkey's egg, to the left of the epigastrium, and apparently attached to the surrounding parts. She was ordered ten grammes of the tincture of eucalyptus daily, with divers interruptions, during five months, and at the end of that time the tumor had become much smaller, isolated and more movable, less firm in consistence, and more sensible to pressure; and at the same time the patient had lost the cachetic appearance, and appeared in better health. During the interruption in the administration of the medicine, the patient's sufferings were increased, and the return to it produced a marked amelioration. She is still under observation.

The third case was that of a man, fifty-three years of age, suffering from a tumor in the right hypochondriac and epigastric regions, and presenting many of the subjective symptoms of cancer of the stomach. After three months' treatment by the tincture of eucalyptus, ten grammes daily, the patient was much better, and the tumor had diminished in size and consistence. He is still under treatment.

The fourth case was that of a man, fifty-two years of age, suffering from cancer of the stomach for ten and a half months, and in a much worse condition than the two preceding. In fact, he was reduced to an incredable state of consumption, by the black vomiting, and was almost moribund. Ten-gramme doses of the tincture of eucalyptus were administered, with great relief to the symptoms for a short time, but the vomiting soon returned, and could only be controlled by subcutaneous injections of sulphate of soda. The patient is still under observation, and Prof. Luton considers that his life has been considerably prolonged by the treatment.

The fifth case was that of a woman, forty-five years of age, suffering from excessive metrorrhagia, uncontrollable by the usual hemostatics, with symptoms of cancer uteri in its early stage. The hemorrhage was completely controlled in less than eight days by ten grammes of the tincture of eucalyptus daily. After the relief of the metrorrhagia, the woman would not submit to an examination, so that there is some doubt about this case.

The sixth case was that of an old gentleman, nearly eighty years of age, who had an epithelial cancer removed, by the knife, from the root of the nose; but the disease subsequently returned, and attacked the nose and the angle of the right eye, producing a large ulcerating tumor, which bled excessively. The first dose of the tincture of eucalyptus produced a sensible effect, and after a very few more the tumor perished and was thrown off, leaving a large excavation with healthy granulations. The process of reparation proceeded most favorably, and was almost completed at the time of publishing the case.

